# Exosomal CircRNA_102619–miR-330-3p Interaction Regulates Osteosarcoma Progression and Metastasis by Forming the Lung Pre-Metastatic Niche

**DOI:** 10.3390/ijms27209193

**Published:** 2026-10-16

**Authors:** Ming-Yu Lien, Yu-Hsiang Chang, Hsiao-Chi Tsai, Chin-Jung Hsu, Chun-Lin Liu, Jeng-Hung Guo, Tsung-Yu Ho, Liang-Wei Lin, Yi-Chin Fong, Chih-Hsin Tang

**Affiliations:** 1 School of Medicine, China Medical University, Taichung 40402, Taiwan; leinmirain@hotmail.com; 2 Division of Hematology and Oncology, Department of Internal Medicine, China Medical University Hospital, Taichung 40447, Taiwan; 3 Program for Cancer Biology and Drug Discovery, China Medical University, Taichung 40402, Taiwan; billy0225@icloud.com; 4 Department of Medicine Research, China Medical University Beigang Hospital, Yunlin 65152, Taiwan; moxa0110@gmail.com; 5 Department of Orthopedic Surgery, China Medical University Hospital, Taichung 40447, Taiwan; jeffrey5991@gmail.com (C.-J.H.); 020394@tool.caaumed.org.tw (T.-Y.H.); 6 School of Chinese Medicine, China Medical University, Taichung 40402, Taiwan; 7 Department of Neurosurgery, China Medical University Hospital, Taichung 40447, Taiwan; chunlin2539@gmail.com (C.-L.L.); 014299@tool.caaumed.org.tw (J.-H.G.); 8 Department of Pharmacology, School of Medicine, China Medical University, Taichung 40402, Taiwan; luce1061@gmail.com; 9 Department of Sports Medicine, College of Health Care, China Medical University, Taichung 40402, Taiwan; 10 Department of Orthopedic Surgery, China Medical University Beigang Hospital, Yunlin 65152, Taiwan; 11 Chinese Medicine Research Center, China Medical University, Taichung 40402, Taiwan; 12 Department of Medical Laboratory Science and Biotechnology, College of Medical and Health Science, Asia University, Taichung 41354, Taiwan

**Keywords:** osteosarcoma, extracellular vesicles, circRNA_102619

## Abstract

Osteosarcoma, the most prevalent primary bone tumor, predominantly influences children and adolescents. The pre-metastatic niche (PMN) is a specialized microenvironment that forms before clinically detectable metastatic lesions. Cancer-associated fibroblasts (CAFs) play a crucial function in triggering lung metastasis by coordinating PMN formation in the lung. We found that circRNA_102619, delivered by osteosarcoma-derived extracellular vesicles (EVs), promotes the reprogramming of lung fibroblasts (LFs) into CAFs and PMN formation both in vitro and in vivo. High-throughput protein array analysis revealed that CXCL10, a critical factor secreted by CAFs, drives osteosarcoma proliferation, migration, and resistance to natural killer (NK) cell cytotoxicity. Additionally, circRNA_102619 acts as a sponge for miR-330-3p, thereby controlling CXCL10 production. Importantly, in vivo studies confirmed that osteosarcoma-derived EVs enhance lung PMN formation, promoting osteosarcoma progression and metastasis. Inhibiting osteosarcoma-derived EVs represents a novel strategy to block osteosarcoma progression and metastasis.

## 1. Introduction

Osteosarcoma is the most common primary malignant bone tumor and typically affects children and adolescents. The reported male-to-female incidence ratio is approximately 1.5–2:1 [1]. With over 3600 new cases and 1720 deaths recorded in the USA in 2020, osteosarcoma has a dismal prognosis [2]. Patients with localized cancer may have a five-year survival rate of about 70%, while those with metastatic disease may have a five-year survival rate of less than 20% [3]. The lung is the most common location of metastasis and the main factor influencing outcome in patients with osteosarcoma [4]. Little is known about the fundamental mechanisms that control osteosarcoma cells’ lung tropism.

A number of intricate processes contribute to tumor metastasis, such as tumor cells entering the bloodstream from the original location, clustering in the bloodstream to evade immune destruction and flow shear forces, and then spreading and colonizing in distant sites [5,6]. The premetastatic niche (PMN) is the specific milieu that develops prior to the development of clinically discernible metastatic lesions. PMNs facilitate tumor cell colonization, which in turn promotes the development of metastases. Cells called fibroblasts are found in connective tissue and are implicated in various vital biological functions, for instance tissue repair and wound healing [5,6]. However, because of the spatiotemporal heterogeneity of fibroblasts in the tumor microenvironment, it has become more widely recognized that cancer-associated fibroblasts (CAFs) have a variety of functions [7]. Through their effects on metabolic reprogramming, matrix breakdown, and angiogenesis, CAFs have been shown in recent studies to be involved in tumor proliferation, invasion, metastasis, and treatment resistance [8,9,10]. More significantly, fibroblasts are a newly emerging population that has the potential to coordinate the incidence and development of PMN during its evolution. Fibroblasts are a very diverse cell type and have been considered a possible therapeutic target to reduce metastasis by modifying PMN production.

Tumor-derived extracellular vesicles (EVs), which are secreted by tumors, serve as essential factors in intercellular communication and are pivotal in the establishment of the PMN and the promotion of organ tropism in metastasis [11]. These nano-sized (30–150 nm) vesicles with a lipid bilayer membrane can encapsulate different cargos from parent cells, transport them to recipient cells, and further promote particular functions [12,13]. There is increasing documentation that tumor-derived EVs play a multifunctional role in immune system modulation, extracellular matrix remodeling, and the induction of angiogenesis or lymphangiogenesis—all of which contribute to tumor metastasis [14,15]. We found that circular RNAs (circRNA)_102619, derived from osteosarcoma EVs, promote the transformation of lung fibroblasts (LFs) into CAFs and the formation of PMN. Subsequently, CAF-secreted C-X-C motif chemokine ligand 10 (CXCL10) enhances osteosarcoma progression and metastasis. Mechanistically, circRNA_102619 acts as a sponge for microRNA (miR)-330-3p, thereby modulating CXCL10 synthesis. Our investigation provides new insights into the molecular mechanisms by which osteosarcoma-derived EVs induce the transformation of LFs into CAFs within the lung PMN, promoting osteosarcoma progression and metastasis.

## 2. Results

### 2.1. Exosomal CircRNA_102619 from Osteosarcoma Facilitates the Transformation of LFs into CAFs and the Formation of the PMN

**Figure 1:**
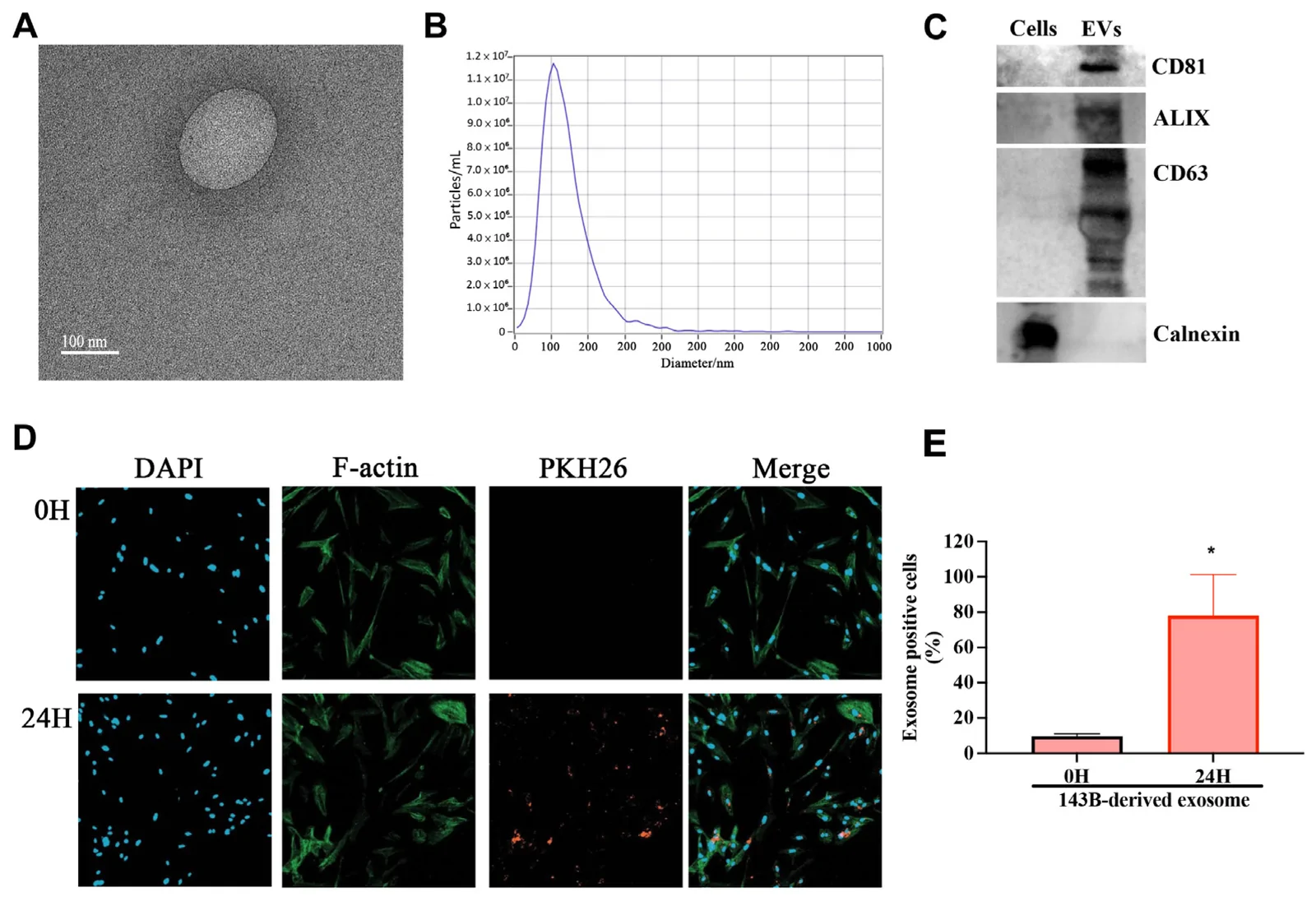
Characterization and uptake of 143B-derived exosomes by IMR-90 cells. (**A**) A transmission electron microscopy (TEM) image showing the typical morphology of 143B-derived extracellular vesicles (EVs). (**B**) Nanoparticle tracking analysis (NTA) indicating the size distribution of isolated EVs, with a predominant peak around 100–150 nm. (**C**) Western blot analysis showing the enrichment of EV markers CD81, ALIX, and CD63, and the absence of the negative marker calnexin in isolated EVs. (**D**) Representative fluorescence images showing the uptake of PKH26-labeled 143B-derived EVs by IMR-90 cells after 24 h. Nuclei were stained with DAPI (blue), F-actin was stained with phalloidin (green), and EVs were labeled with PKH26 (red). (**E**) Quantification of PKH26-positive IMR-90 cells after incubation with 143B-derived EVs for 0 or 24 h. All experiments were independently repeated three times with similar results. * *p* < 0.05 compared with the control group.

**Figure 2:**
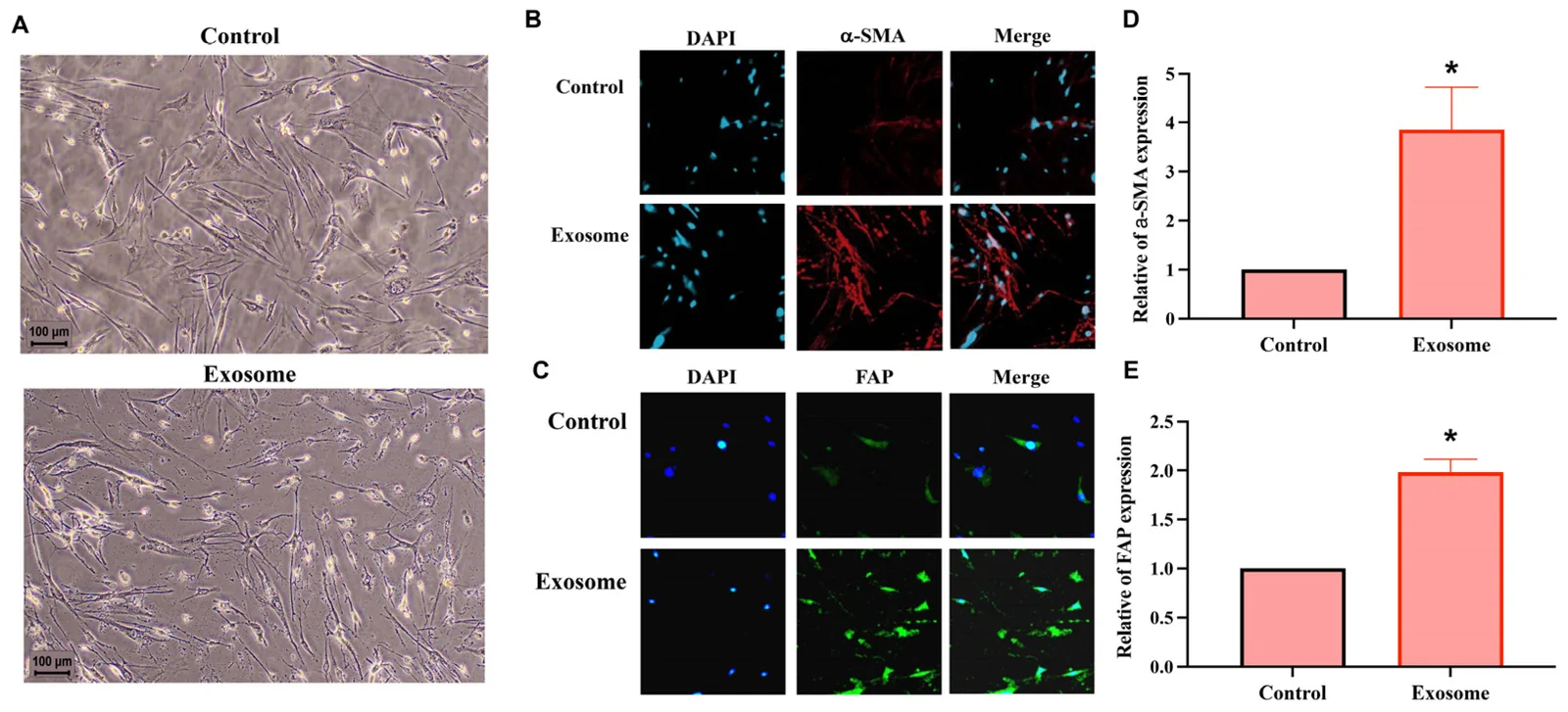
Osteosarcoma-derived EVs induce fibroblast activation and CAF marker expression in IMR-90 cells. (**A**) Representative phase-contrast images showing morphological changes of IMR-90 cells following 24 h incubation with 143B-derived EVs. Exosome-treated cells exhibited a more stretched, spindle-like morphology compared to controls. Scale bar: 100 μm. (**B**,**C**) Immunofluorescence staining showed α-SMA (red, (**B**)) and FAP (green, (**C**)) expression in control and exosome-treated IMR-90 cells, with nuclei counterstained using DAPI (blue). (**D**,**E**) Quantitative analysis of α-SMA (**D**) and FAP (**E**) fluorescence intensity. All experiments were independently repeated three times with similar results. * *p* < 0.05 compared with the control group.

Tumor-derived EVs have the potential to rewire LFs and encourage PMN formation [16]. They are a crucial conduit of communication between primary tumors and metastatic locations [11]. To investigate their potential role in osteosarcoma-mediated reprogramming of fibroblasts, we isolated and characterized EVs from the 143B osteosarcoma cell line. TEM and NTA confirmed the typical morphology and size distribution of these vesicles (Figure 1A,B). Western blot analysis confirmed the enrichment of EV markers CD81, ALIX, and CD63, whereas the negative marker calnexin was absent in EVs (Figure 1C). To assess the uptake capacity of LFs for EVs, we labeled EVs with PKH26 and incubated them with human LF IMR-90 cells. Fluorescence microscopy revealed significantly increased EV uptake by IMR-90 cells after 24 h (Figure 1D,E). The transition to CAFs included morphological changes, such as a more stretched, spindle-like morphology in EV-treated cells (Figure 2A). Additionally, the CAF markers α-SMA and FAP were upregulated, as confirmed by immunofluorescence staining (Figure 2B–E), indicating that osteosarcoma-derived EVs facilitate the reprogramming of LFs into CAFs. Exosomal circRNAs have been implicated in activating CAF functions [12].

**Figure 3:**
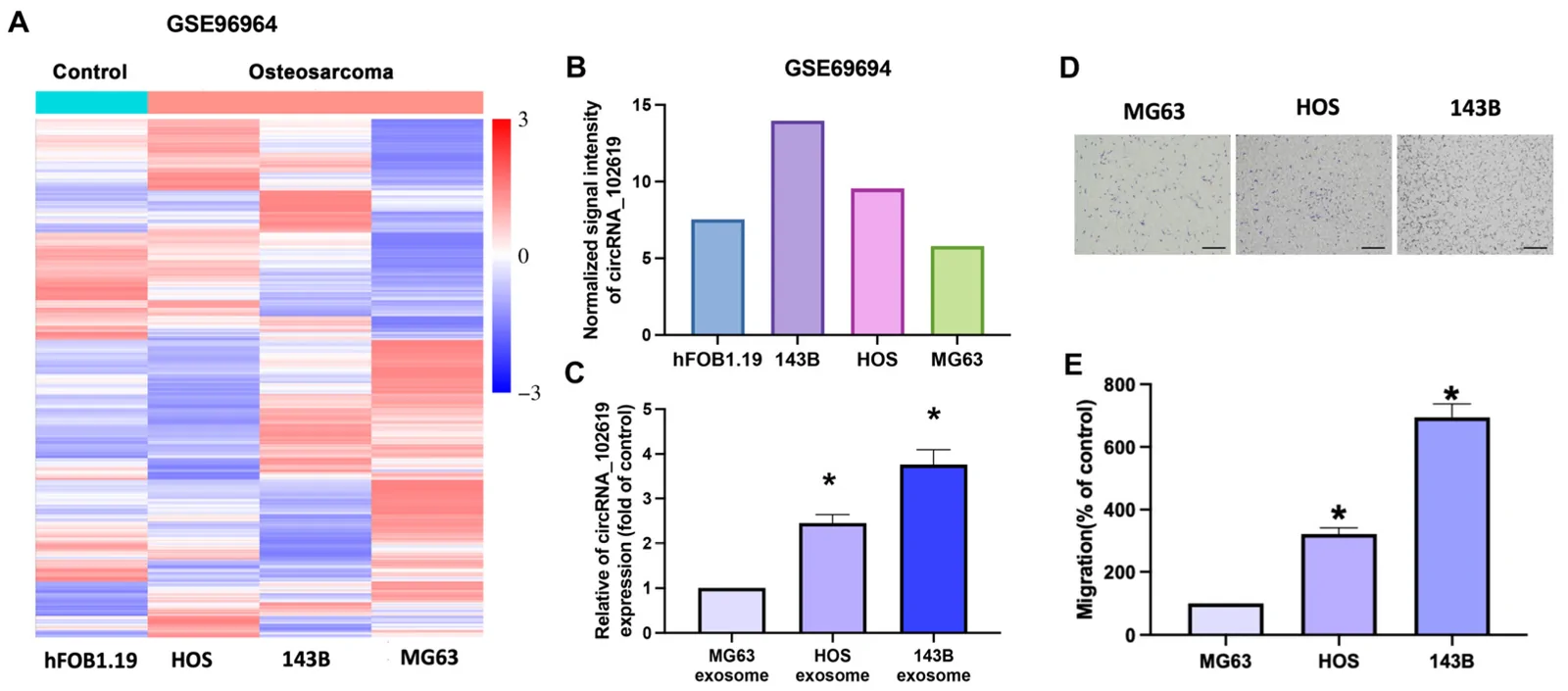
circRNA_102619 is highly expressed in osteosarcoma cells and their derived exosomes, and correlates with osteosarcoma cell migration ability. (**A**) Heatmap analysis of circRNA expression profiles derived from the GSE96964 dataset, comparing osteosarcoma cells (MG63, HOS, and 143B) to osteoblasts (hFOB1.19). (**B**) Expression levels of circRNA_102619 in hFOB1.19 MG63, HOS, and 143B cells based on the GSE96964 dataset. (**C**) Quantitative RT-PCR analysis showing elevated circRNA_102619 expression in exosomes derived from MG63, HOS, and 143B cells. Data are presented as fold change relative to MG63 exosomes. (**D**) Representative images of transwell migration assays demonstrating the migration ability of MG63, HOS, and 143B cells. Scale bar: 100 μm. (**E**) Quantification of cell migration ability. All experiments were independently repeated three times with similar results. * *p* < 0.05 compared with MG63 cells.

To identify candidate circRNAs potentially involved in EV-mediated reprogramming of CAFs, we analyzed publicly available circRNA microarray data from the GSE96964 dataset. Global circRNA expression profiling revealed distinct expression patterns among the osteoblast cell line hFOB1.19 and the osteosarcoma cell lines MG63, HOS, and 143B (Figure 3A). Among the candidate circRNAs, circRNA_102619 exhibited heterogeneous expression across these cell lines. We found that 143B cells exhibited the highest circRNA_102619 expression, followed by HOS and hFOB1.19 cells, whereas MG63 cells showed the lowest expression (Figure 3B). We therefore further examined circRNA_102619 in EVs isolated from the three osteosarcoma cell lines. qPCR analysis demonstrated that EVs from osteosarcoma cells revealed higher circRNA_102619 expression in 143B cells and their EVs compared to HOS and MG63 cells and their EVs, which correlated with their migratory ability (Figure 3C–E), suggesting that circRNA_102619 is a critical mediator in osteosarcoma-derived EVs.

### 2.2. CXCL10 Is a Critical Factor Secreted from CAFs

**Figure 4:**
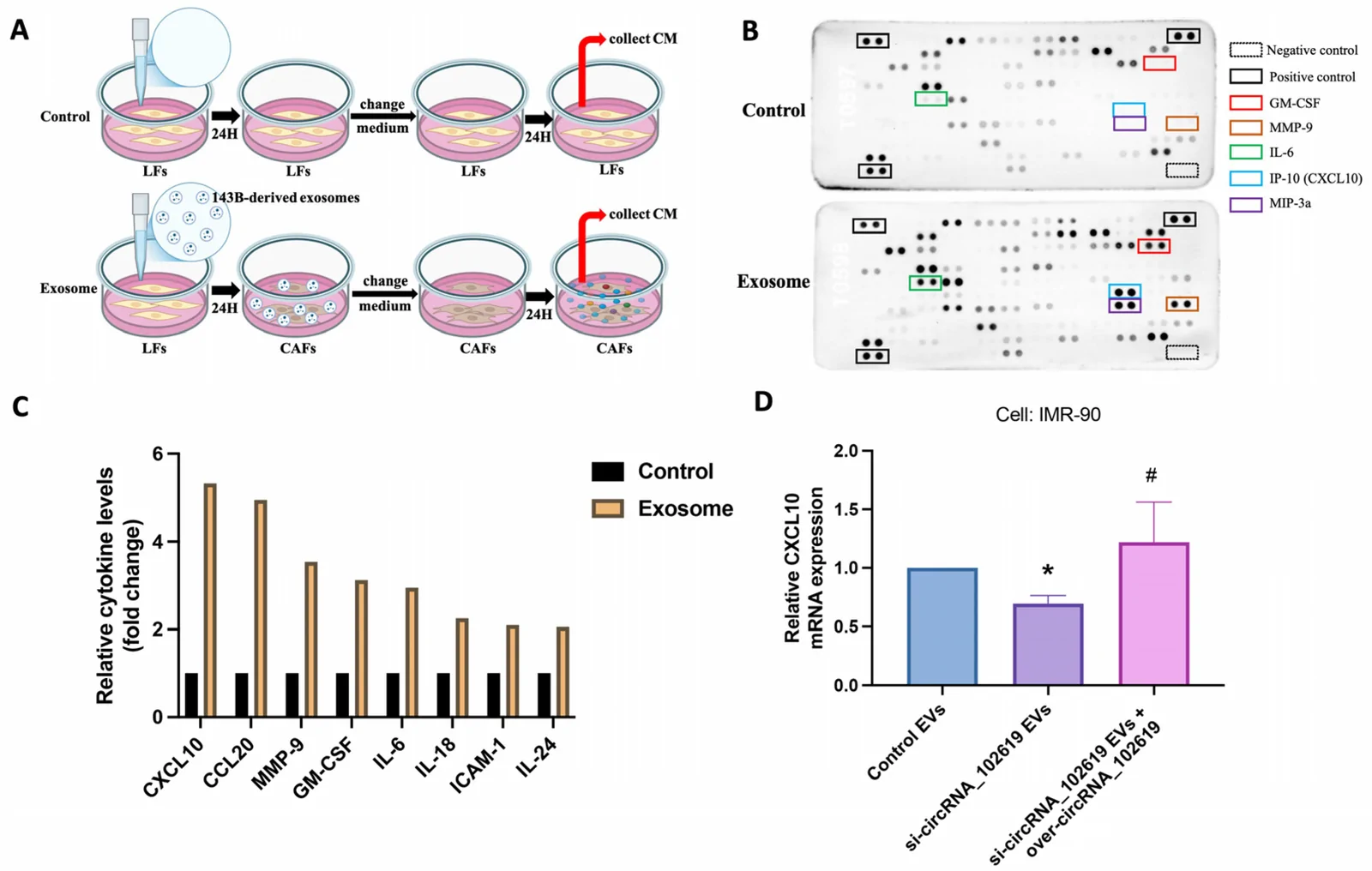
Exosomal circRNA_102619 regulates CXCL10 secretion from EV-induced CAFs. (**A**) A schematic diagram of the experimental procedure. Conditioned medium (CM) was collected from IMR-90 lung fibroblasts (LFs) treated with or without 143B-derived exosomes for 24 h. Created in BioRender.com (web-based version). Tang, C. (2026) https://BioRender.com/bhy5a4d. (**B**) High-throughput protein array analysis of CM obtained from control and exosome-treated fibroblasts. Differentially expressed cytokines are highlighted, including GM-CSF, MMP-9, IL-6, IP-10 (CXCL10), and MIP-3a. (**C**) Quantitative analysis of selected upregulated cytokines based on protein array data. Signal intensities were quantified using ImageJ software (version 1.53k; National Institutes of Health, Bethesda, MD, USA), and fold changes were calculated relative to the control group, which was set to 1. The complete cytokine expression profile is provided in Supplementary Table S2. (**D**) Quantitative RT-PCR analysis showing decreased CXCL10 mRNA expression in IMR-90 fibroblasts treated with exosomes derived from circRNA_102619 siRNA-transfected 143B cells, which was restored by direct overexpression of circRNA_102619 in IMR-90 fibroblasts. All experiments were independently repeated three times with similar results. * *p* < 0.05 compared with the control group. # *p* < 0.05 compared with the si-circRNA_102619 EV group.

**Figure 5:**
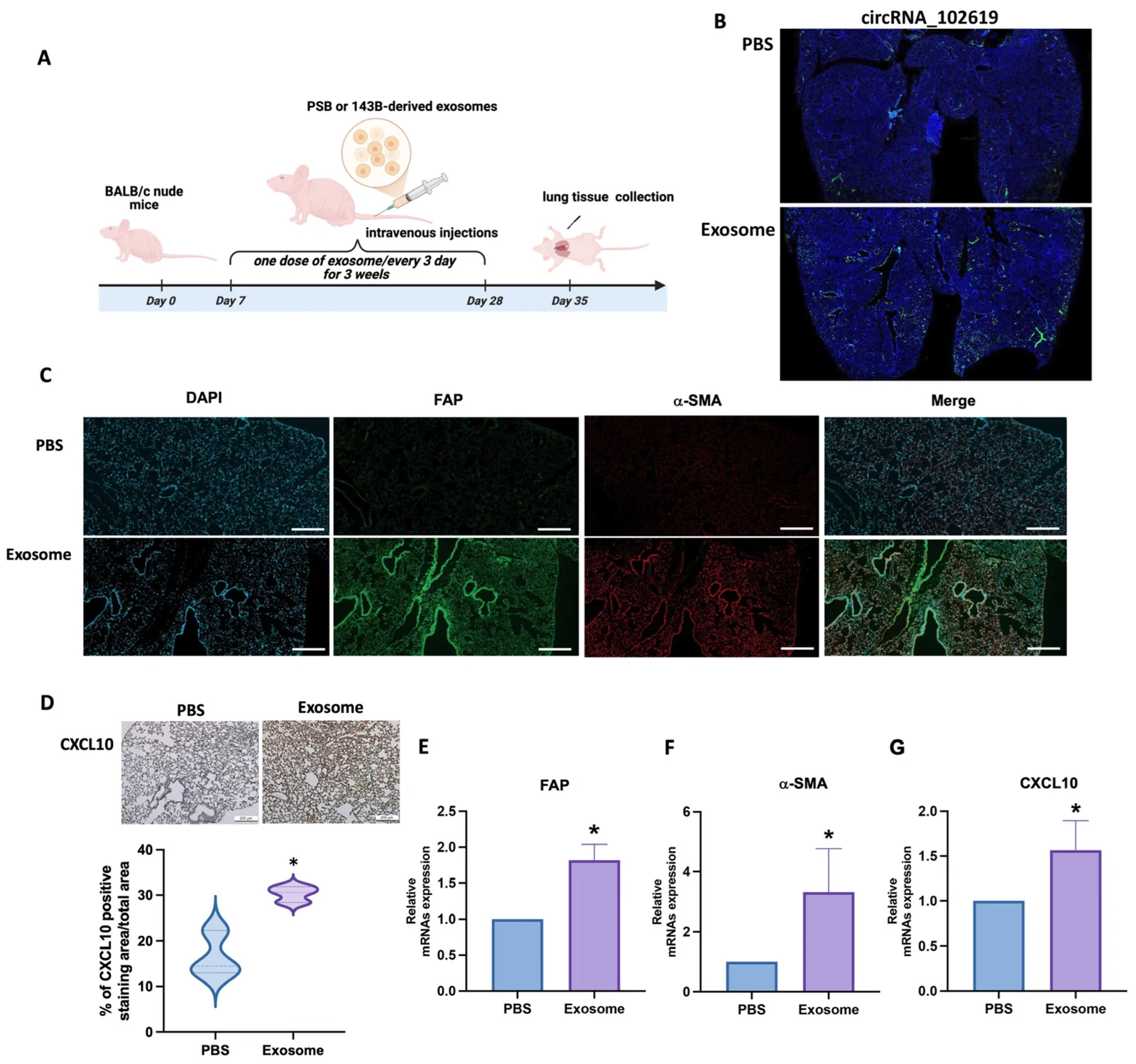
Exosomal circRNA_102619 promotes CAF activation and premetastatic niche (PMN) formation in vivo. (**A**) A schematic illustration of the in vivo tumor education model. BALB/c nude mice were intravenously injected with 143B-derived exosomes (Exosome group) or PBS (Control group) every 3 days for 3 weeks. Lung tissues were harvested for subsequent analyses. Created in BioRender.com (web-based version). Tang, C. (2026) https://BioRender.com/cr5jrq3. (**B**) Representative fluorescence images showing circRNA_102619 signals (green) in lung tissues from PBS- and EV-treated mice, with DAPI counterstaining (blue). (**C**) Representative immunofluorescence images of lung tissue sections stained for fibroblast activation protein (FAP, green) and α-smooth muscle actin (α-SMA, red), with DAPI counterstaining (blue). Scale bar: 100 μm. (**D**) Representative immunohistochemistry images showing CXCL10 expression in lung tissues (upper panel), and quantification of the CXCL10-positive staining area (lower panel). (**E**–**G**) Quantitative RT-PCR analysis of FAP (**E**), α-SMA (**F**), and CXCL10 (**G**) mRNA expression in lung tissues. All experiments were independently repeated three times with similar results. * *p* < 0.05 compared with the control group.

To identify potential factors secreted by CAFs, we collected CM from LFs and EV-induced CAFs (Figure 4A). High-throughput protein array analysis demonstrated elevated levels of GM-CSF, IL-6, MIP-3α (CCL20), MMP-9, and CXCL10, among which CXCL10 exhibited the most pronounced increase (Figure 4B,C). Quantitative analysis revealed that CXCL10 exhibited the highest fold change among the upregulated cytokines (Figure 4C). The complete cytokine array quantification data, including relative fold changes, are provided in Supplementary Table S2. The knockdown efficiency of circRNA_102619 was first confirmed by qPCR (Supplementary Figure S1). EVs derived from circRNA_102619-silenced osteosarcoma cells significantly reduced CXCL10 mRNA expression by 30% in IMR-90 LFs compared with EVs from control siRNA-transfected cells (Figure 4D). Furthermore, overexpression of circRNA_102619 in IMR-90 restored CXCL10 expression (Figure 4D), supporting a regulatory role of osteosarcoma-derived exosomal circRNA_102619 in CXCL10 expression in fibroblasts. We next demonstrated that EVs from osteosarcoma promote PMN formation in vivo. Mice were injected with osteosarcoma-derived EVs for three weeks (Figure 5A). Fluorescence probe analysis revealed increased circRNA_102619 signals in lung tissues from EV-treated mice compared with controls (Figure 5B). Lung tissues from these mice expressed significantly higher levels of α-SMA, FAP, and CXCL10 compared to controls (Figure 5C–G). These results suggest that osteosarcoma-derived EVs are associated with increased pulmonary circRNA_102619 expression, CAF-associated marker expression, and PMN formation.

### 2.3. CircRNA_102619 Acts as a Sponge for miR-330-3p, Subsequently Promoting CXCL10-Dependent Osteosarcoma Progression and Metastasis

**Figure 6:**
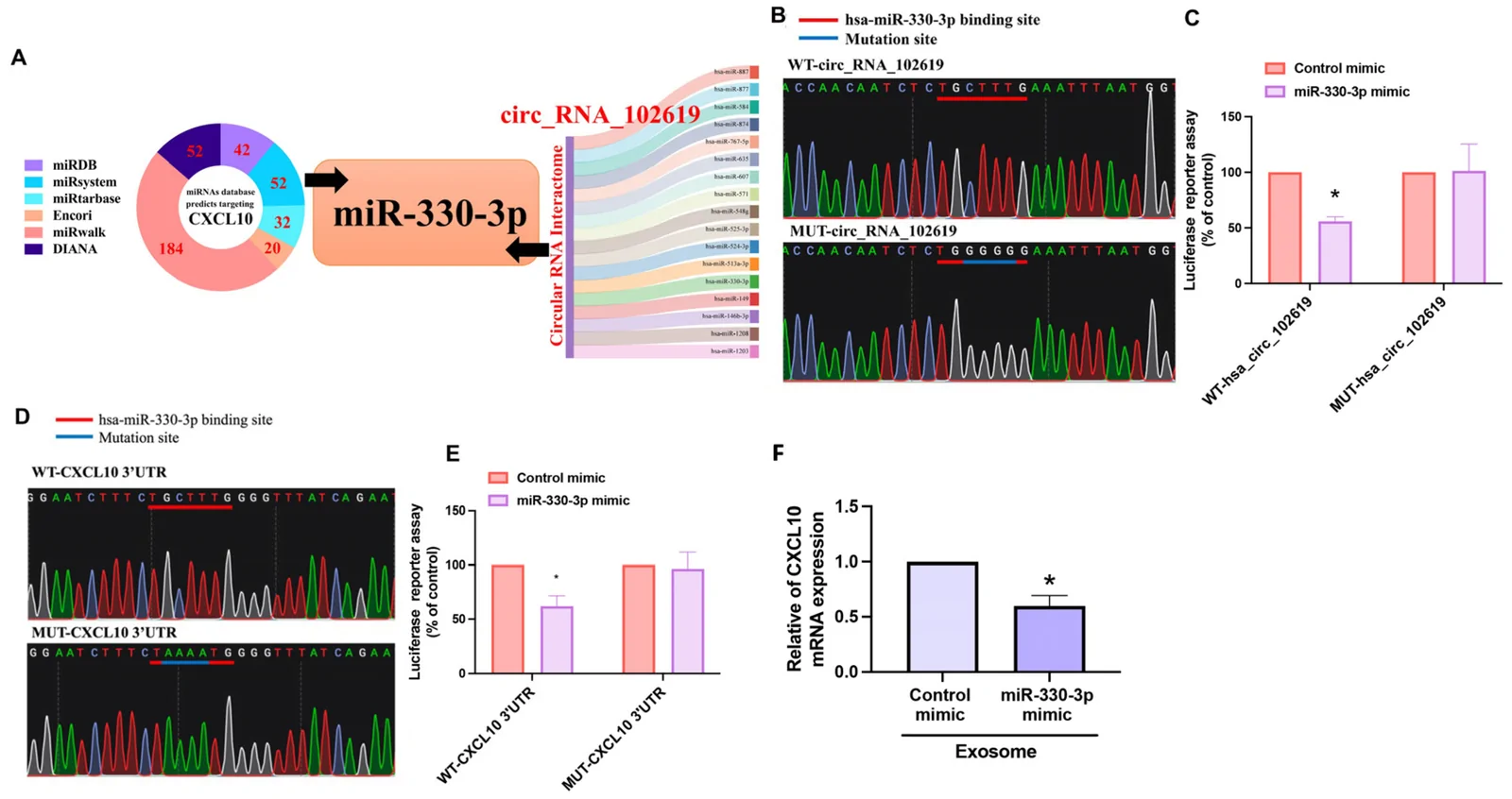
CircRNA_102619 regulates CXCL10 expression via sponging miR-330-3p. (**A**) Integrated bioinformatics analysis using six miRNA databases (miRDB, miRSystem, miRTarbase, EncorI, miRwalk, DIANA) and the Circular RNA Interactome identified miR-330-3p as the common candidate targeting both circRNA_102619 and CXCL10. (**B**) Sanger sequencing confirmed the wild-type (WT) and mutant (MUT) binding sites for miR-330-3p on circRNA_102619. (**C**) Dual-luciferase reporter assays showed that the miR-330-3p mimic suppressed luciferase activity of WT circRNA_102619 reporter but not the mutant reporter. (**D**) Sanger sequencing confirmed WT and MUT binding sites for miR-330-3p on CXCL10 3′-UTR. (**E**) Dual-luciferase reporter assays demonstrated that the miR-330-3p mimic decreased the luciferase activity of WT CXCL10 3′-UTR but not of MUT CXCL10 3′-UTR. (**F**) Quantitative RT-PCR analysis showed that the miR-330-3p mimic reduced CXCL10 mRNA expression in exosome-treated fibroblasts. All experiments were independently repeated three times with similar results. * *p* < 0.05 compared with the control group.

**Figure 7:**
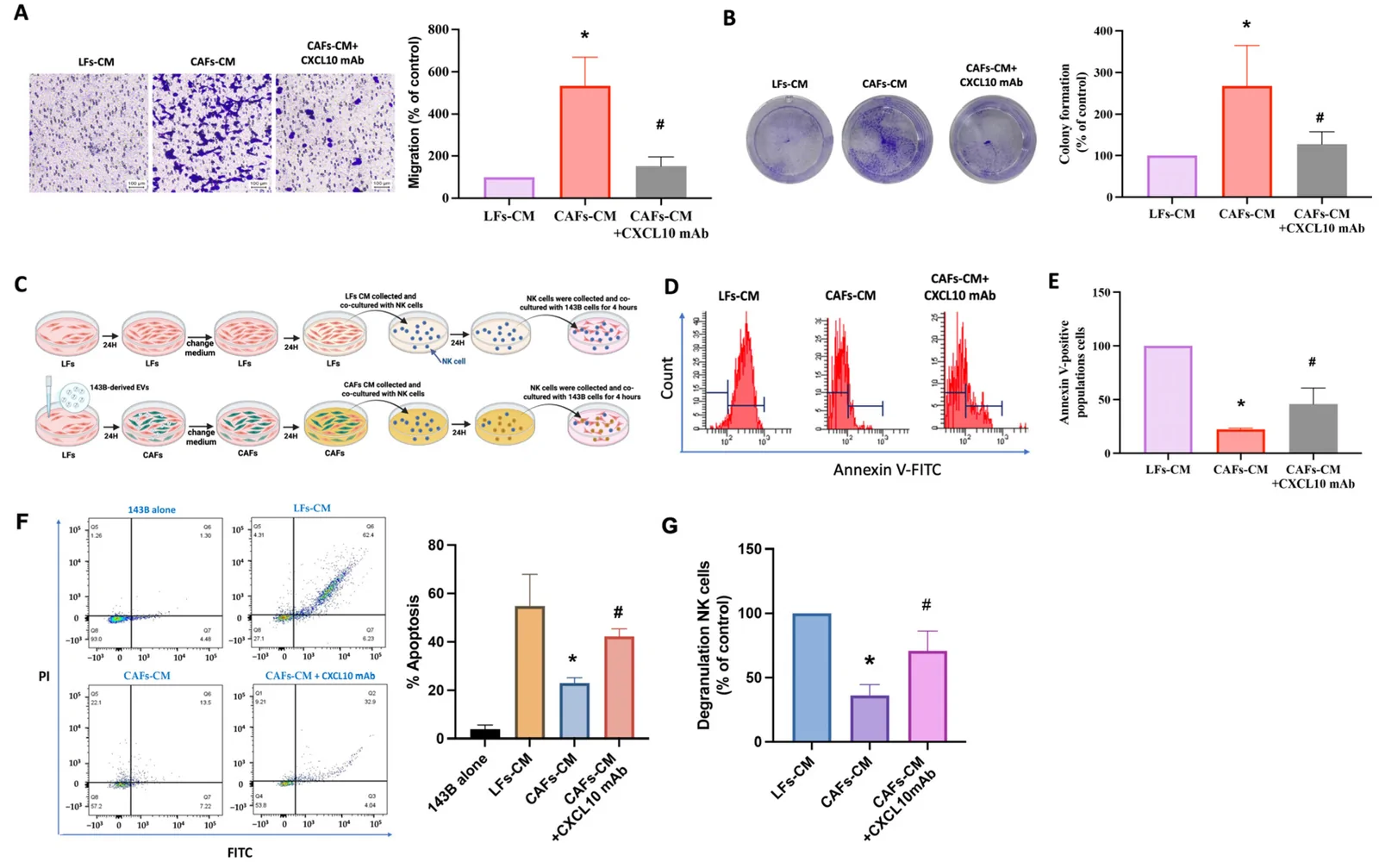
EV-induced CAF-derived CM promotes osteosarcoma cell migration, colony formation, and resistance to NK cell-mediated cytotoxicity via CXCL10. (**A**) Cell migration assay showing enhanced migration of 143B osteosarcoma cells treated with CM from EV-induced CAFs (CAFs-CM) compared to LFs-CM. The pro-migratory effect was significantly reduced by treatment with a CXCL10 monoclonal antibody (mAb). (**B**) A colony formation assay showing increased clonogenic ability of 143B cells treated with CAF-CM, which was attenuated by CXCL10 mAb. (**C**) A schematic of the experimental design: LFs were treated with 143B-derived EVs to generate CAFs. CM was collected from LFs or EV-induced CAFs and used to stimulate NK cells. NK cells were subsequently co-cultured with 143B cells for 4 h. Created in BioRender.com (web-based version). Tang, C. (2026) https://BioRender.com/pv5kmr1. (**D**,**E**) Flow cytometry analysis showing reduced Annexin V-positive populations in 143B cells co-cultured with CAF-CM-treated NK cells, which was partially reversed by CXCL10 mAb treatment. (**F**) Representative Annexin V/PI flow cytometry plots and quantification of 143B cell apoptosis following co-culture with NK cells pretreated with different types of CM. NK cells were excluded using an NK cell-specific marker to selectively analyze 143B cell apoptosis. (**G**) Flow cytometry analysis of CD107a expression showing reduced NK cell degranulation following CAF-CM treatment, which was partially restored by CXCL10 mAb. All experiments were independently repeated three times with similar results. * *p* < 0.05 compared with the LF-CM group; # *p* < 0.05 compared with the CAF-CM group.

CircRNAs act as competing endogenous RNAs by sponging miRNAs, thereby preventing their repression of target genes [17]. To investigate whether circRNA_102619 regulates CXCL10 expression through miRNA interactions, we analyzed six miRNA databases to predict miRNA binding to CXCL10 and identified candidates commonly predicted by all six databases. These candidates were further intersected with miRNAs predicted to bind circRNA_102619 using the Circular RNA Interactome tool, identifying miR-330-3p as the only overlapping candidate (Figure 6A). Transfection with an miR-330-3p mimic inhibited luciferase activity of WT circRNA_102619 and WT CXCL10 3′-UTR constructs as well as CXCL10 mRNA expression but not MT circRNA_102619 and MT CXCL10 3′-UTR constructs lacking miR-330-3p binding sites (Figure 6B–F), indicating that miR-330-3p directly binds to circRNA_102619 and CXCL10. To further investigate the cellular functions of activated CAFs [18], we compared CM from LFs with CM from EV-induced CAFs. CM from EV-induced CAFs promoted osteosarcoma cell migration and colony formation (Figure 7A,B), effects that were blocked by CXCL10 mAb. Additionally, NK cells pretreated with CM from EV-induced CAFs were co-cultured with osteosarcoma cells. Annexin V/PI flow cytometry revealed reduced apoptosis in 143B cells co-cultured with CAF-CM-treated NK cells, which was partially restored by CXCL10 mAb treatment (Figure 7C–F). Furthermore, CAF-CM significantly reduced NK cell degranulation, whereas CXCL10 neutralization partially reversed this effect (Figure 7G). These results indicate that osteosarcoma-derived EVs promote CAF activation, inducing osteosarcoma proliferation, migration, and resistance to NK cell-mediated cytotoxicity through CXCL10.

### 2.4. Osteosarcoma-Derived EVs Educate the Lung PMN, Thereby Facilitating Osteosarcoma Progression and Metastasis In Vivo

**Figure 8:**
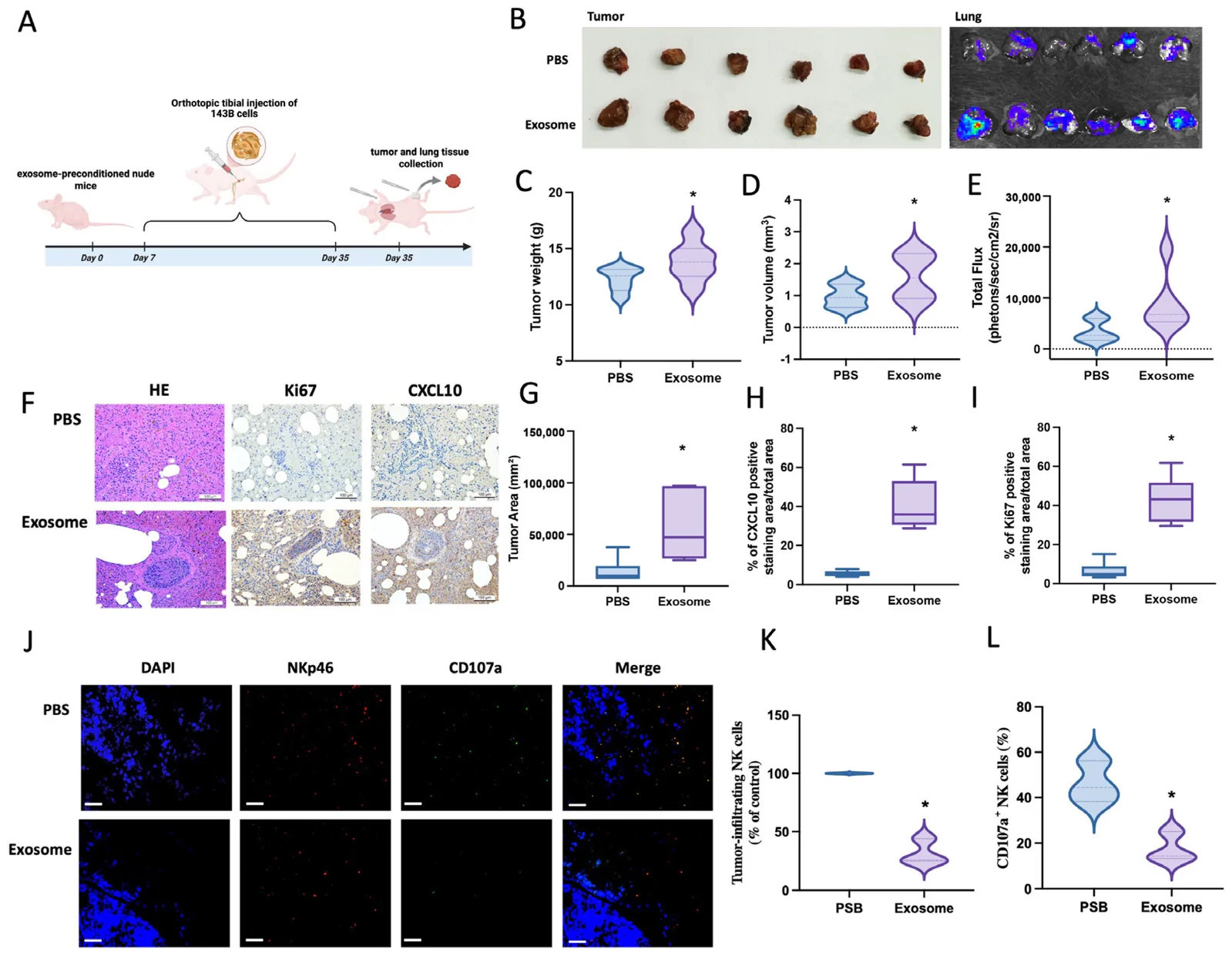
Osteosarcoma-derived exosomes promote tumor progression, lung metastasis, and NK cell suppression in vivo. (**A**) A schematic illustration of the orthotopic osteosarcoma mouse model. Created in BioRender.com (web-based version). Tang, C. (2026) https://BioRender.com/3zv5uqa. (**B**) Representative images of primary tumors (left) and IVIS imaging of lung metastases (right) from PBS- and exosome-treated mice. (**C**) Tumor weight of primary tibial tumors. (**D**) Tumor volume of primary tibial tumors. (**E**) Total lung metastasis bioluminescence signals detected by IVIS imaging. (**F**) Representative H&E and immunohistochemical (IHC) staining for Ki67 and CXCL10 in lung tissues. (**G**–**I**) Quantification of the number of tumor area (**G**), Ki67-positive area (**H**), and CXCL10-positive area (**I**) in lung tissues. (**J**) Representative immunofluorescence staining of tumor-infiltrating NK cells (NKp46 and CD107a) in lung tissues. Scale bar: 100 μm. (**K**,**L**) Quantification of tumor-infiltrating NKp46^+^ NK cells (**K**) and CD107a^+^ NK cells (**L**). All experiments were independently repeated three times with similar results. * *p* < 0.05 compared with the control group.

To investigate whether osteosarcoma-derived EVs educate the lung PMN and promote osteosarcoma progression and metastasis in vivo, we established an orthotopic mouse model. Mice were orthotopically implanted with 143B osteosarcoma cells in the right tibia, followed by intravenous injection of osteosarcoma-derived EVs for three weeks, with tumor growth monitored using the IVIS system (Figure 8A). After 4 weeks, tumors in the tibia were larger in the EV-educated group compared to the control group (Figure 8B–D). IVIS imaging further revealed increased lung metastasis in the EV-treated group (Figure 8B,E). IHC analysis showed significantly elevated levels of Ki67 and CXCL10 in the lung tissues of the EV-treated group (Figure 8F–I). Additionally, reduced expression of NK cell markers NKp46 and CD107a was found in the EV-educated group (Figure 8J–L). These findings indicate that osteosarcoma-derived EVs promote lung PMN formation, thereby enhancing osteosarcoma progression and metastasis in vivo.

## 3. Discussion

One of the most aggressive types of cancer found in youngsters is osteosarcoma. Because of the sequential complete therapy of preoperative chemotherapy, postoperative chemotherapy, and tumor resection, the five-year survival rate for localized osteosarcoma can currently exceed 60% [19]. Nevertheless, pulmonary metastases become the primary cause of osteosarcoma-related mortality, lowering the survival rate of osteosarcoma patients to less than 33% of those without metastases [20]. One essential prerequisite for the pulmonary metastasis of osteosarcoma is the remodeling of the local lung milieu into the PMN, which promotes the colonization and growth of tumor cells [21,22]. We discovered that the osteosarcoma EV-derived circRNA_102619 stimulates the development of the PMN and the conversion of LFs into CAFs. Consequently, CXCL10 released by CAF promotes the growth and metastasis of osteosarcoma. Mechanistically, circRNA_102619 controls the production of CXCL10 by acting as a sponge for miR-330-3p. Therefore, targeting circRNA_102619, the PMN, and CXCL10 represents a potential strategy for inhibiting osteosarcoma progression and metastasis.

There are several phases in the creation of the PMN, but the participation of primary tumor-secreted factors and the remodeling of the extracellular matrix milieu at metastatic locations are given special attention [23,24]. One of the most important processes during PMN development is the activation of fibroblasts into CAFs [24]. According to recent research, LFs may be reprogrammed to support PMN formation by tumor-secreted EVs, a crucial conduit of communication between primary tumors and metastatic locations [11]. Tumor-derived EVs have organ-specific targeting due to different integrin expression, and lung-targeted EVs and LFs co-localize in the pulmonary milieu [25]. Our results demonstrate that LFs uptake osteosarcoma-derived EVs, promoting their reprogramming into CAFs, as evidenced by upregulated production of the CAF markers α-SMA and FAP. Interestingly, our in vivo data further confirm that osteosarcoma-derived EVs enhance CAF activation and PMN formation. Therefore, EV-mediated tumor–lung communication represents a potential target for inhibiting lung PMN formation. Previous studies classified HOS and HOS-143B as having high migratory and invasive potential, whereas MG63 exhibited low activity in both assays based on a systematic characterization of 22 osteosarcoma cell lines [26]. Furthermore, 143B displayed the greatest migratory activity among the related parental TE-85/HOS and derivative MNNG/HOS and 143B cell lines [27]. These findings support the higher migratory capacity of HOS and 143B compared with MG63, as well as the enhanced migratory phenotype of 143B relative to its parental cell line. In our study, circRNA_102619 levels were higher in 143B-derived than in MG63-derived EVs, consistent with their greater migratory capacity. In addition, Hou et al. showed that CXCL14 depletion reduced HOS cell migration without altering proliferation, further demonstrating that osteosarcoma cell motility is influenced by specific molecular programs [28].

Our array findings indicate that osteosarcoma-derived EVs alter a broader fibroblast secretory program rather than selectively increasing CXCL10. The concomitant increases in IL-6 and GM-CSF may be relevant to stromal–immune interactions. A recent study demonstrated that CAF-derived IL-6 promoted macrophage polarization toward an M2-like phenotype and impaired antitumor immunity, with IL-6 receptor blockade alleviating these effects [29]. GM-CSF also regulates myeloid cell differentiation and function, although its effects in cancer are context-dependent and can support either antitumor immunity or tumor-associated immunosuppression [30]. The increased CCL20 and MMP-9 signals additionally suggest potential changes in chemokine-mediated cell recruitment and extracellular matrix remodeling. CCL20 acts through CCR6 and has been implicated in the recruitment of immune cell populations, including Th17 cells, as well as tumor progression in several cancer settings. These effects are dependent on the cellular and tumor context [31]. MMP-9 is a matrix-remodeling protease rather than a cytokine, and its relevance to osteosarcoma is supported by a 2024 study in which MMP9 downregulation reduced tumor growth and lung metastatic burden in an orthotopic model [32]. However, these effects are context-dependent and remain to be established in osteosarcoma.

CXCL10 was prioritized in this study because it showed a prominent increase in the array and its induction in recipient fibroblasts was attenuated by circRNA_102619 knockdown in donor osteosarcoma cells. Recent osteosarcoma-specific evidence supports a pro-migratory role for the CXCL10–CXCR3 axis. Exogenous CXCL10 enhanced migration in 143B, LM7, and MG63.3 cells and increased AKT and PAK1 phosphorylation [33]. Genetic deletion of CXCR3 reduced tumor cell migration and proliferation, primary tumor growth, and pulmonary metastasis in an orthotopic model; re-expression of CXCR3A, but not CXCR3B, restored aggressive phenotypes [33]. These findings provide a plausible receptor-mediated context for the migration-promoting effects associated with fibroblast-derived CXCL10 in our study. However, the same report found that exogenous CXCL10 did not increase proliferation, and pharmacological CXCR3 inhibition reduced lung metastasis without significantly affecting primary tumor growth. Interestingly, the magnitude of the change in CXCL10 secretion was greater than that observed at the mRNA level. Such discordance between cytokine transcript and protein levels is not unexpected, as cytokine production is regulated at multiple levels, including mRNA stability, translation, protein processing, and secretion [34]. Indeed, CXCL10 has been reported to undergo post-transcriptional regulation through RNA-binding proteins and signaling-dependent modulation of mRNA stability [35,36].

CircRNAs are a unique class of RNA that, through a special back-splicing mechanism during RNA splicing, adopt a closed-loop architecture [37]. This creates a class of extremely stable RNAs that are hard to diminish and may have a crucial effect on a number of biological roles [38]. By vying for miRNA binding sites, circRNA acts as a miRNA sponge, lessening the impact of miRNA-mediated regulatory actions [39]. There is evidence linking circRNA to metastasis and carcinogenesis [40]. The cancer-related functions of circRNA_102619 remain poorly understood. A previous study identified circRNA_102619 (circNOL10) as an upregulated candidate in non-small-cell lung cancer but did not investigate its function [41]. Our study extends these findings by demonstrating the involvement of osteosarcoma EV-derived circRNA_102619 in intercellular communication with lung fibroblasts. In this study, we also examined six miRNA databases to predict miRNA binding to CXCL10 and used the Circular RNA Interactome tool to predict miRNA binding to circRNA_102619 in order to determine whether circRNA_102619 controls CXCL10 expression through miRNA interactions. The only candidate that was found was miR-330-3p. Recent evidence supports a role for miR-330-3p in restraining malignant phenotypes in osteosarcoma. It has been found that circ_0078767 promoted osteosarcoma proliferation, invasion, and migration through the miR-330-3p/CDK14 axis, and that miR-330-3p counteracted these circRNA-associated effects [42]. This study establishes a precedent for circRNA-dependent regulation of miR-330-3p in osteosarcoma, but does not connect miR-330-3p to CXCL10 or lung fibroblast activation. Here, we provide evidence that miR-330-3p binds directly to circRNA_102619 and CXCL10. Thus, circRNA_102619, by sponging miR-330-3p, is critical for regulating CXCL10 expression.

Several limitations should be acknowledged. First, GSE96964 revealed heterogeneous circRNA_102619 expression across osteosarcoma cell lines, with higher levels in 143B and HOS but lower levels in MG63 compared with hFOB1.19. Further validation in additional cell lines and clinical specimens is warranted. Second, GSE96964 reflects cellular circRNA expression, which may not directly correspond to EV-associated circRNA levels. Third, although increased circRNA_102619 signals were observed in a lung tissue animal model following EV treatment, further in vivo knockdown studies are required to establish its specific contribution to PMN formation. Fourth, although the wild-type/mutant dual-luciferase reporter assays and functional experiments support a regulatory interaction between circRNA_102619 and miR-330-3p, these approaches do not directly demonstrate their endogenous physical interaction. Therefore, whether circRNA_102619 functions as an endogenous sponge for miR-330-3p should be further validated using RNA pull-down or AGO2-RIP assays. Although our findings support the proposed circRNA_102619–miR-330-3p–CXCL10 axis, further investigation of RNA abundance, binding site availability, and subcellular localization is needed to establish the physiological relevance of the endogenous miRNA sponge mechanism. Moreover, the specific contribution of CXCL10 to fibroblast-mediated tumor progression should be distinguished from that of other secreted factors, particularly given their potentially divergent effects on tumor cell migration and proliferation. Because CXCR3 signaling affects both tumor and immune cells, further studies are warranted to evaluate its therapeutic implications in the context of antitumor immunity.

**Figure 9:**
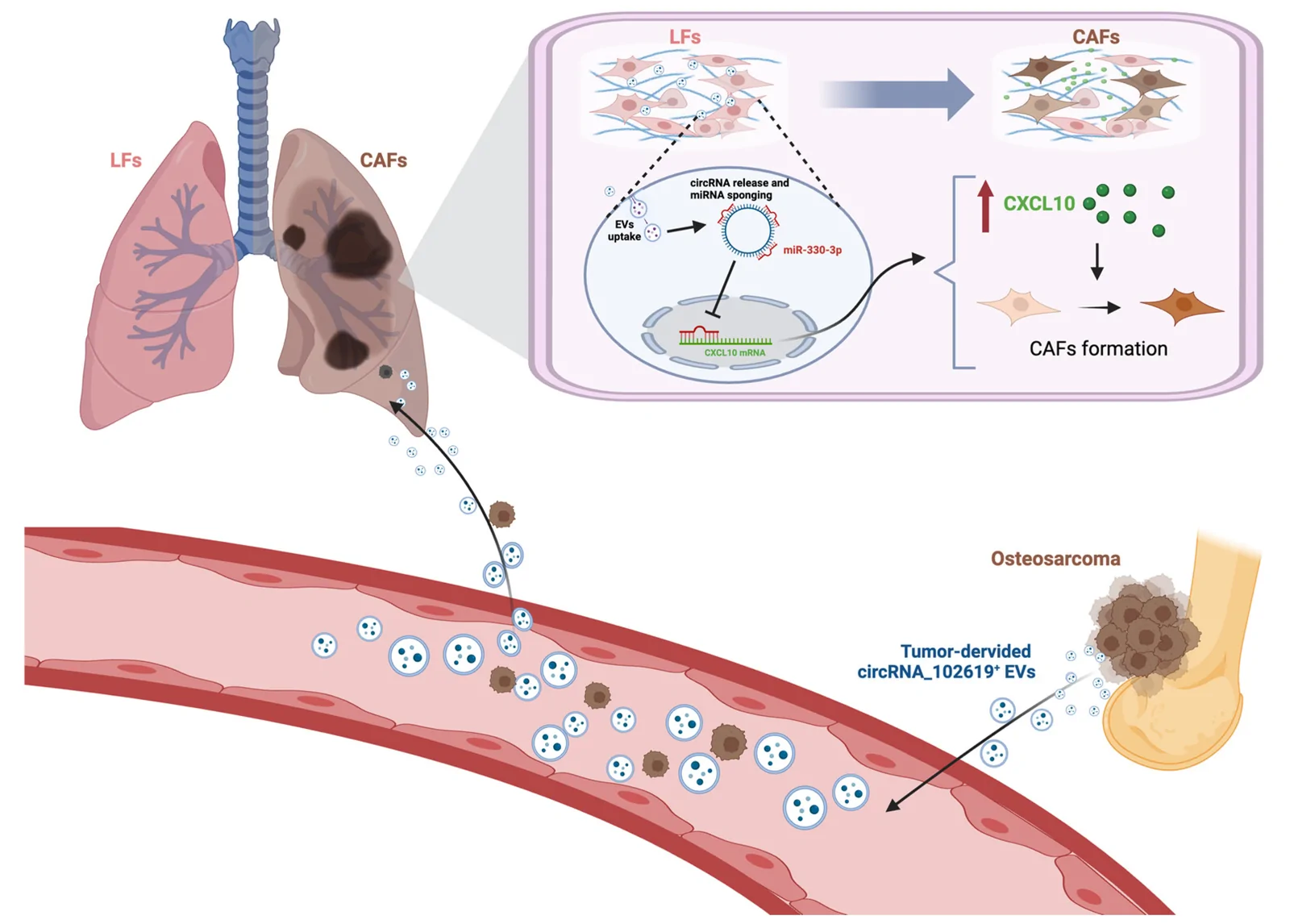
A schematic diagram illustrating the mechanism underlying the roles of osteosarcoma-derived EVs in the progression, metastasis and NK cell immune escape of osteosarcoma. Osteosarcoma-derived EVs deliver circRNA_102619 to lung fibroblasts, promoting their reprogramming into CAFs. CircRNA_102619 sponges miR-330-3p and increases CXCL10 production, which enhances osteosarcoma proliferation and migration while reducing NK cell-mediated cytotoxicity. Together, these effects promote lung premetastatic niche formation, immune escape, and metastasis. Created in BioRender.com (web-based version). Tang, C. (2026) https://BioRender.com/8p4onty.

In summary, we discovered that circRNA_102619, delivered by osteosarcoma-derived EVs, promotes lung PMN formation and the reprogramming of LFs into CAFs. CXCL10, a critical factor secreted by CAFs, drives osteosarcoma growth and metastasis both in vitro and in vivo. Mechanistically, circRNA_102619 regulates CXCL10 expression by sponging miR-330-3p (Figure 9).

## 4. Materials and Methods

### 4.1. Materials

Total Exosome Isolation Reagent (Cat# 4478359), TRIzol reagent (Cat# 15596026), Lipofectamine 3000 (Cat# L3000008) and DAPI (Cat# D1306) were obtained from Invitrogen, Thermo Fisher Scientific (Waltham, MA, USA). PKH26 dye (Cat# PKH26GL-1KT) and crystal violet (Cat# C0775) were obtained from Sigma-Aldrich (St. Louis, MO, USA). PrimeScript RT reagent kit (Cat# RR037A) was obtained from Takara (Shiga, Japan). The Annexin V-FITC/PI apoptosis detection kit (Cat# 556547) was obtained from BD Biosciences (Franklin Lakes, NJ, USA). The Proteome Profiler Human Cytokine Array Kit (Cat# ARY022B) was obtained from R&D systems (Minneapolis, MN, USA). Anti-Ki67 (Cat# ab15580) and anti-CXCL10 antibody (Cat# ab25191) were obtained from Abcam (Cambridge, UK). Anti-FAP antibody (Cat# 66562) was obtained from Cell signaling (Danvers, MA, USA). Anti-CD63 (Cat# A5271) and anti-ALIX (PDCD6IP; Cat# A2215) antibodies were obtained from ABclonal. Anti-CD81 antibody (Cat# GTX101766) was obtained from GeneTex. Anti-calnexin antibody (Cat# sc-23954) was obtained from Santa Cruz Biotechnology. Anti-mouse CD107a (Cat# 121606) and anti-mouse NKp46 (Cat# 137608), FITC-conjugated anti-human CD107a (LAMP-1; clone H4A3) and APC-conjugated anti-human NKp46 (CD335; clone 9E2) antibodies were obtained from BioLegend (San Diego, CA, USA). The Novolink Max Polymer Detection System (Cat# RE7280-K) was obtained from Leica Biosystems (Wetzlar, Germany). The dual-luciferase reporter assay system (Cat# E1910) was obtained from Promega (Madison, WI, USA).

### 4.2. Cell Culture

Human osteosarcoma cell lines (143B [RRID: CVCL_2270], HOS [RRID: CVCL_1284], and MG63 [RRID: CVCL_0426]) and the human lung fibroblast cell line IMR-90 (RRID: CVCL_0347) were obtained from the Bioresource Collection and Research Center (BCRC, Hsinchu, Taiwan). The human natural killer (NK) cell line NK-92MI (RRID: CVCL_4859) was purchased from the American Type Culture Collection (ATCC, Manassas, VA, USA). Osteosarcoma and lung fibroblast cells were cultured in DMEM supplemented with 10% FBS, 100 U/mL penicillin, and 100 μg/mL streptomycin. NK-92MI cells were cultured in α-MEM supplemented with 12.5% FBS, 12.5% horse serum, 2 mM L-glutamine, 0.2 mM inositol, 0.1 mM 2-mercaptoethanol, 0.02 mM folic acid, and 100 U/mL penicillin–streptomycin. Cells were maintained at 37 °C in a humidified incubator containing 5% CO_2_. All cell lines were authenticated by the suppliers and tested negative for mycoplasma contamination before use.

### 4.3. Exosome Isolation and Characterization

Exosomes were isolated from 143B cell-conditioned medium (CM) using the Total Exosome Isolation Reagent according to the manufacturer’s instructions. Briefly, 20 mL of cell culture supernatants was collected, centrifuged at 2000× *g* for 30 min to remove cell debris, and filtered through a 0.22 μm filter. The exosome precipitation reagent was applied, and samples were incubated overnight at 4 °C. Exosomes were subsequently pelleted by centrifugation at 10,000× *g* for one hour. The isolated exosomes were resuspended in 100 μL of PBS, and protein concentrations were determined using a bicinchoninic acid (BCA) assay before storage at −80 °C until use. Exosome morphology was examined by transmission electron microscopy (TEM), and size distribution was analyzed by nanoparticle tracking analysis (NTA). To further characterize the isolated exosomes, Western blot analysis was performed to assess the expression of the exosomal markers CD81, ALIX, and CD63, as well as the negative marker calnexin. Protein lysates from parental 143B cells and isolated exosomes were analyzed to confirm the enrichment of exosomal markers and the absence of detectable calnexin in the exosome preparations.

### 4.4. Protein Array Analysis of CM

CM from control IMR-90 fibroblasts and EV-induced CAFs was collected after 24 h. Cytokine profiles were analyzed using the Proteome Profiler Human Cytokine Array Kit (R&D Systems) according to the manufacturer’s instructions. Equal volumes of CM from each group were processed in parallel. Chemiluminescent signals were captured, and the spot intensities were quantified by densitometric analysis using ImageJ software (National Institutes of Health, Bethesda, MD, USA). For each analyte, duplicate spots were averaged after local background subtraction and normalized to the internal positive-control spots on the corresponding membrane.

### 4.5. Exosome Uptake Assay

Exosomes that had been purified were marked with PKH26 dye following the manufacturer’s instructions. Exosomes labeled with PKH26 were used to incubate IMR-90 cells for 24 h. Uptake was visualized by fluorescence microscopy with 4′,6-diamidino-2-phenylindole (DAPI, nuclear stain) and fibroblast activation protein (FAP). Quantification of exosome internalization was performed using ImageJ software.

### 4.6. Bioinformatic Analysis

Publicly available circRNA microarray data were retrieved from the Gene Expression Omnibus (GEO) database (GSE96964). The dataset included circRNA expression profiles of osteosarcoma cell lines (MG63, HOS, and 143B) and osteoblasts (hFOB1.19). The processed expression values provided by GEO, which had undergone quantile normalization, were used for subsequent analysis. Subsequent prediction of miRNA binding sites was performed using Circular RNA Interactome (https://circinteractome.nia.nih.gov/(accessed on 17 September 2026)), and six miRNA target prediction databases (miRDB, miRSystem, miRTarBase, EncorI, miRWalk, DIANA) were utilized to identify potential miRNAs binding to CXCL10.

### 4.7. Flow Cytometric Analysis of Tumor Cell Apoptosis and NK Cell Degranulation

NK-92MI cells were pretreated for 24 h with conditioned medium from lung fibroblasts (LF-CM) or EV-induced cancer-associated fibroblasts (CAF-CM), with or without a CXCL10-neutralizing monoclonal antibody. Following pretreatment, NK-92MI cells were collected and co-cultured with 143B cells at an effector-to-target ratio 4:1 of for 4 h [43]. This 4 h incubation period was selected to assess NK cell-mediated cytotoxicity during short-term effector–target interactions [43]. 143B cells cultured without NK-92MI cells served as the control. Cells were collected and stained with Annexin V-FITC and propidium iodide (PI) using the Annexin V-FITC/PI apoptosis detection kit according to the manufacturer’s instructions. Samples were analyzed using a BD FACSLyric (BD Biosciences, San Jose, CA, USA), and data were processed using FlowJo_v10.8.1. 143B cells were distinguished from NK-92MI cells using an APC-conjugated anti-human NKp46 (CD335). Apoptosis was quantified as the percentage of Annexin V-positive cells, including Annexin V-positive/PI-negative and Annexin V-positive/PI-positive cells, within the gated 143B population.

NK-92MI cells were pretreated with the indicated conditioned media for 24 h and subsequently co-cultured with 143B cells at an effector-to-target ratio 4:1 of for 4 h, as described above. Surface CD107a was detected using FITC-conjugated anti-human CD107a antibody. The antibody was added during co-culture. NK-92MI cells were identified using APC-conjugated anti-human NKp46 (CD335). Degranulation was quantified as CD107a mean fluorescence intensity. Values were normalized to the LF-CM group, which was set to 100%. Samples were acquired using a BD FACSLyric and analyzed using FlowJo_v10.8.1.

### 4.8. Immunofluorescence Staining of NK Cells

For evaluation of NK cell infiltration and degranulation, frozen lung sections were stained with antibodies against NKp46 and CD107a, and nuclei were counterstained with DAPI. NKp46^+^ and CD107a^+^ cells were quantified by fluorescence microscopy.

### 4.9. Real-Time Quantitative PCR (RT-qPCR)

Using TRIzol reagent according to the manufacturer’s instructions, total RNA was extracted from cells or tissues. First-strand cDNA was synthesized using the MMLV RT reagent kit (Invitrogen). Quantitative polymerase chain reaction (PCR) was performed using SYBR Green Master Mix (Thermo Fisher Scientific) on a StepOnePlus Real-Time PCR system (Applied Biosystems, Foster City, CA, USA). The following target genes were analyzed: circRNA_102619, CXCL10, alpha-smooth muscle actin (α-SMA), and FAP. 18S and Glyceraldehyde-3-phosphate dehydrogenase (GAPDH) were used as internal controls. Relative gene expression levels were calculated using the 2^−ΔΔCt^ method. All reactions were performed in triplicate [44,45]. The primer sequences used for RT-qPCR analysis are listed in Supplementary Table S1.

### 4.10. Dual-Luciferase Reporter Assay

Wild-type (WT) and mutant (MUT) sequences of circRNA_102619 and CXCL10 3′-UTR containing predicted miR-330-3p binding sites were cloned into luciferase reporter vectors. 143B cells were co-transfected with reporter plasmids and the miR-330-3p mimic or control mimic. Luciferase activity was investigated using a dual-luciferase assay system [46,47].

### 4.11. Cell Migration and Colony Formation Assays

The Boyden chamber migration assay was used to evaluate the migration of osteosarcoma cells. Serum-free medium was used to seed cells in the upper chamber, while CM was introduced into the lower chamber. After a 24 h period, the migrated cells were stained with crystal violet and subsequently counted using a microscope. For the colony formation assay, cells were seeded in 24-well plates at a density of 1000 cells per well and cultured for 14 days, with the medium refreshed every 3 days. Afterwards, colonies were fixed with 4% paraformaldehyde for 15 min at room temperature and stained with 0.1% crystal violet for 20 min. Visible colonies were photographed, and the number of colonies (defined as clusters containing more than 50 cells) was quantified using ImageJ software (National Institutes of Health, Bethesda, MD, USA) by applying brightness/contrast thresholding and the “Analyze Particles” tool [48,49].

### 4.12. Histology and Immunohistochemistry

Lung tissues were fixed in 10% formalin, embedded in paraffin, and sectioned at 4 μm thickness for hematoxylin and eosin (H&E) staining and immunohistochemical (IHC) analysis. IHC staining was assessed using the Novolink Max Polymer Detection System (Leica Biosystems) according to the manufacturer’s protocols. Primary antibodies against Ki67 and CXCL10 were applied. The positive staining areas were quantified using ImageJ software [50,51].

### 4.13. In Vivo PMN Formation and Lung Metastasis Model

Four-week-old male BALB/c nude mice (National Laboratory Animal Center, Taiwan) were randomly assigned to control and exosome-treated groups (*n* = 6 per group). The individual mouse was considered the experimental unit. Mice were housed under a 12 h light/dark cycle in standard cages with corncob bedding and provided food and water ad libitum. Prior to exosome or osteosarcoma cell injection, mice were anesthetized in a closed chamber with 3% isoflurane in 100% oxygen for 3–5 min and then transferred to a heating pad maintained at 36.5 °C to minimize pain and distress during injection. Survival and health status were monitored daily. Mice received intravenous injections of 143B-derived exosomes (100 µg/mouse) or PBS every 3 days for 3 weeks. Lung tissues were subsequently harvested for histological and molecular analyses. After completion of PMN formation, 143B osteosarcoma cells (5 × 10^5^ cells/mouse) were orthotopically implanted into the right tibia under anesthesia. Four weeks after tumor implantation, mice were sacrificed, and lung tissues were collected for further histological and immunofluorescence staining. The investigators were blinded to group allocation during both histological and immunofluorescence analyses.

All animal experiments were conducted in accordance with institutional and international ethical standards, including the guidelines of the International Council for Laboratory Animal Science (ICLAS), and were performed following the Animal Research: Reporting of In Vivo Experiments (ARRIVE) guidelines. Sample sizes were estimated based on prior experience and the published literature to achieve sufficient statistical power to detect the expected effects. The study was approved by the Institutional Animal Care and Use Committee (IACUC) of China Medical University (Protocol No. CMUIACUC-2025-132). According to the IACUC-approved protocol, mice were sacrificed if tumor burden exceeded 10% of body weight, if tumor growth impaired mobility, or if weight loss exceeded 25% from baseline. No animals exceeded this limit during the course of the experiment.

### 4.14. Statistical Analysis

All experiments were performed at least in triplicate. No samples or data points were excluded from the analysis. All experimental replicates were included unless technical errors (e.g., contamination or equipment malfunction) occurred, in which case the experiment was repeated. Data are presented as the mean ± standard deviation (SD). Statistical significance was determined using Student’s *t*-test or one-way ANOVA, with *p* < 0.05 considered statistically significant.

## Data Availability

The original data generated in this study are available from the corresponding authors upon reasonable request. Publicly available datasets analyzed in this study are accessible through the repositories and accession numbers cited in the manuscript.

## Supplementary Materials

The following supporting information can be downloaded at: https://www.mdpi.com/article/10.3390/ijms27209193/s1.

## Abbreviations

The following abbreviations are used in this manuscript:

α-SMA	Alpha-smooth muscle actin
CAFs	Cancer-associated fibroblasts
circRNAs	Circular RNAs
CM	Cell-conditioned medium
CXCL10	C-X-C motif chemokine ligand 10
DAPI	4′,6-diamidino-2-phenylindole
EVs	Extracellular vesicles
FAP	Fibroblast activation protein
GAPDH	Glyceraldehyde-3-phosphate dehydrogenase
IHC	Immunohistochemical
LFs	Lung fibroblasts
miR	MicroRNA
NK	Natural killer
NTA	Nanoparticle tracking analysis
PCR	Polymerase chain reaction
PMN	Premetastatic niche
TEM	Transmission electron microscopy
