# Supplementary material for: Exosomal CircRNA_102619–miR-330-3p Interaction Regulates Osteosarcoma Progression and Metastasis by Forming the Lung Pre-Metastatic Niche
Source: Int J Mol Sci. 2026 Oct 16;27(20):9193. doi: 10.3390/ijms27209193 (PMC13704919; doi:10.3390/ijms27209193)
Supplement: Supplementary file 1 [file ijms-27-09193-s001.zip › ijms-4604703-supplementary.pdf]

Supplementary Materials

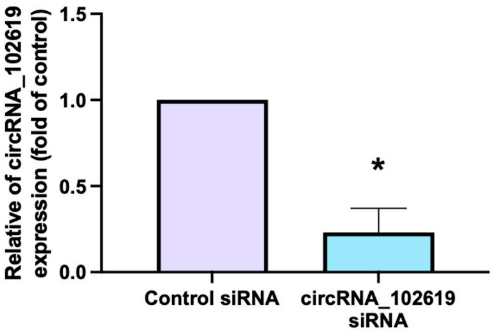

Figure S1. Knockdown efficiency of circRNA_102619. The expression levels of circRNA_102619 in 143B osteosarcoma cells transfected with negative control siRNA or circRNA_102619 siRNA were determined by RT-qPCR. Data are presented as the mean ± SD. **P* < 0.05 versus control siRNA.

Supplementary Tables

Table S1. Primer sequences used for RT-qPCR analysis.

Primer sequences are presented in the 5′–3′ direction.

Target	Forward primer (5′–3′)	Reverse primer (5′–3′)
circRNA_102619	GGGCATCTATTACATTCCATTCT	ATTATTCTCCGCAGCATCAGT
18S	CCAGTAAGTGCGGGTCATAAG	GGCCTCACTAAACCATCCAA
CXCL10	GTGGCATTCAAGGAGTACCTC	TGATGGCCTTCGATTCTGGATT
FAP	TGAACGAGTATGTTTGCAGTGG	GGTCTTTGGACAATCCCATGT
ACTA2 (α-SMA)	CTATGCCTCTGGACGCACAACT	CAGATCCAGACGCATGATGGCA
GAPDH	ACCACAGTCCATGCCATCAC	TCCACCACCCTGTTGCTGTA

Abbreviations: RT-qPCR, reverse transcription quantitative polymerase chain reaction; FAP, fibroblast activation protein; ACTA2, actin alpha 2, smooth muscle; GAPDH, glyceraldehyde-3-phosphate dehydrogenase.

Table S2. Cytokine array profile.

Complete quantitative profile of cytokines detected by protein array analysis in conditioned medium from control fibroblasts and EV-induced cancer-associated fibroblasts.

Cytokine / protein	Control spot 1	Control spot 2	Exosome spot 1	Exosome spot 2	Original plotted log2FC	Array spot pair
IP-10 (CXCL10)	2.8879	3.3329	124.4529	124.2229	5.3285	F19-20
MIP-3α (CCL20)	4.0975	3.3905	113.4434	115.3770	4.9476	G19-20
MMP-9	5.6642	11.3853	84.8828	93.9546	3.5384	G23-24
GM-CSF	8.9398	10.3669	80.4294	88.0127	3.1282	C21-22
IL-6	9.9117	10.1028	81.2702	73.3783	2.9507	E5-6
IL-18 Bpa	5.0866	6.5553	22.7061	33.1453	2.2510	E23-24
ICAM-1	5.3994	4.8245	23.0991	20.6825	2.0985	D7-8
IL-24	4.1617	3.0927	15.4580	14.2343	2.0560	F7-8
LIF	7.3198	3.2690	13.0505	20.2852	1.9979	G3-4
IL-23	3.9648	1.9096	9.1730	10.6858	1.9836	F5-6
Dkk-1	20.8931	22.7676	82.2746	84.9445	1.9390	B15-16
G-CSF	9.4252	9.3439	34.7951	35.6330	1.9079	C17-18
uPAR	14.9397	13.8664	48.7541	47.9478	1.7487	I19-20
TNF-α	2.7203	3.3508	9.8790	8.5529	1.6285	I17-18
IL-22	4.4735	1.7984	5.4306	8.5587	1.5784	F3-4
M-CSF	6.4345	7.9326	21.6408	20.4220	1.5699	G11-12
IL-15	3.2923	7.6922	11.3850	14.3982	1.4141	E17-18
Lipocalin-2	4.2098	3.9602	12.1482	9.4860	1.4008	G5-6
IL-8	43.9824	45.9330	120.8799	111.8334	1.3738	E7-8
Angiopoietin-2	10.9985	11.3337	29.1965	26.4494	1.3185	A11-12

IL-33	1.8942	1.7125	4.1693	4.6474	1.2972	F15-16
ENA-78	42.1022	41.7349	102.9592	102.5730	1.2937	C3-4
TFF3	4.8726	4.4854	10.6949	11.9784	1.2826	I9-10
CD31	7.8250	5.6443	15.4665	16.2302	1.2786	J7-8
RBP-4	4.2211	4.2548	8.5402	11.7352	1.2574	H17-18
IL-17A	3.3726	4.5288	8.2056	10.2081	1.2287	E21-22
Pentraxin 3	37.2244	34.4394	83.1539	84.2436	1.2265	H9-10
IL-11	3.4247	4.2897	8.9483	8.6249	1.2090	E11-12
MCP-1	31.8104	30.7670	72.3918	71.3822	1.2003	G7-8
IL-32	2.7296	1.7101	4.2409	4.7401	1.1128	F13-14
Thrombospondin-1	17.9356	15.9913	36.2381	36.7901	1.1114	I15-16
FGF-7	15.9986	16.5954	35.3503	34.7936	1.1064	C11-12
I-TAC	5.2083	3.1697	6.9845	9.2925	1.0951	F21-22
IGFBP-2	12.9623	14.4842	28.4903	28.2788	1.0532	D11-12
RANTES	3.6368	4.4238	7.6477	8.9943	1.0482	H15-16
IGFBP-3	24.8275	24.5002	49.8997	49.9745	1.0178	D13-14
Endoglin	19.1574	19.9602	38.3217	40.6373	1.0130	C5-6
Relaxin-2	4.3377	4.0036	7.6079	8.9568	0.9968	H19-20
IL-16	3.0982	2.8583	6.3992	5.4685	0.9923	E19-20
TfR	13.6346	11.6119	25.1587	24.7079	0.9902	I11-12
GROα	16.3028	14.4392	30.5593	30.1692	0.9869	D1-2
IL-1α	4.6925	4.8571	8.4102	10.2421	0.9638	D15-16
TGF-α	5.7508	5.5625	9.3483	12.1031	0.9265	I13-14
MCP-3	23.8324	24.0538	48.3994	42.1897	0.9202	G9-10
IL-12 p70	3.6004	2.3732	5.0426	5.5671	0.9055	E13-14

Emmprin	48.0153	49.4504	87.2037	92.7358	0.8842	B21-22
VCAM-1	28.2947	28.0771	51.3392	50.4696	0.8528	J11-12
SHBG	5.5445	5.9841	10.6446	9.9360	0.8401	I3-4
Flt-3 Ligand	8.1753	7.6372	13.9839	13.8208	0.8156	C15-16
Osteopontin	6.5893	5.7194	10.0890	11.0664	0.7933	H3-4
IL-34	2.7022	4.2307	5.1699	6.5132	0.7877	F17-18
IL-13	2.6434	2.7714	5.5948	3.6872	0.7853	E15-16
IL-27	3.0941	4.0597	5.8124	6.0190	0.7490	F9-10
TARC	5.6754	6.1065	10.5564	9.0654	0.7418	I7-8
IL-31	2.3836	3.4748	4.7492	4.5444	0.7226	F11-12
VEGF	72.9009	74.2940	120.2522	120.9164	0.7124	I21-22
PF4	5.2531	5.8698	8.4326	9.6162	0.6976	H11-12
TIM-3	6.0689	7.7153	9.3512	12.8996	0.6838	J9-10
ST2	9.3889	10.5875	14.5946	17.5351	0.6829	I5-6
PDGF-AA	3.7469	6.6765	6.6789	8.9691	0.6443	H5-6
PDGF-AB/BB	5.8669	5.7168	8.5746	9.2351	0.6215	H7-8
Angiopoietin-1	17.3237	18.3361	29.8871	23.3437	0.5842	A9-10
IL-19	6.1828	2.8034	5.7565	5.7844	0.5823	F1-2
Chitinase 3-like 1	51.1016	60.2569	77.0839	83.0166	0.5292	B5-6
IL-1ra	5.2412	6.5101	8.4761	7.9809	0.5075	D19-20
MIF	15.2632	14.4799	19.4813	21.4534	0.4636	G13-14
IL-2	5.7749	8.8629	8.7602	10.9720	0.4620	D21-22
HGF	101.1360	98.6309	133.5868	132.4120	0.4132	D5-6
Growth Hormone	6.1953	5.1264	7.0673	7.7655	0.4090	D3-4
IL-1β	4.0010	4.9979	5.1726	6.6261	0.3888	D17-18

Vitamin D BP	11.2883	8.7574	11.3623	13.6519	0.3592	J5-6
FGF-19	5.4383	6.2777	6.6890	8.2455	0.3468	C13-14
RAGE	6.3321	5.2475	7.7931	6.6264	0.3182	H13-14
Resistin	11.6238	13.0199	13.9693	16.7968	0.3172	H21-22
Serpin E1	91.7914	90.5954	109.2134	114.6757	0.2961	I1-2
CD40 ligand	5.6689	7.4396	7.1782	8.1674	0.2413	B3-4
Fas Ligand	5.5971	4.7063	6.3407	5.4259	0.1927	C7-8
GDF-15	63.0489	72.1860	77.0293	76.0223	0.1858	C19-20
Kallikrein 3	6.2426	6.5805	5.9606	8.6839	0.1855	F23-24
MIG	3.0855	3.0784	3.5677	3.3386	0.1640	G15-16
FGF basic	5.3036	4.8845	5.6646	5.4089	0.1213	C9-10
IL-3	12.8672	11.7822	13.3451	13.1356	0.1057	D23-24
IFN-γ	5.1965	4.8229	5.8418	4.8059	0.0845	D9-10
EGF	9.2775	9.1086	9.7134	9.4113	0.0567	B19-20
IL-10	3.8088	3.4875	2.9052	4.3418	0.0056	E9-10
SDF-1α	25.7693	27.2125	25.8126	27.1887	0.0006	H23-24
Angiogenin	82.2882	80.7861	78.8520	81.0944	-0.0276	A7-8
MIP-1α/MIP-1β	5.0842	2.5541	2.8709	3.5001	-0.0476	G17-18
C-Reactive Protein	6.1167	6.5183	5.3219	6.1582	-0.1402	B9-10
DPPIV	108.2747	100.4400	91.1543	96.9610	-0.1462	B17-18
IL-4	7.3917	5.8666	5.3163	6.2547	-0.1638	E1-2
IL-5	5.7130	5.5909	4.7851	4.9102	-0.2211	E3-4
CD30	9.0420	16.5842	9.3939	10.6662	-0.2498	A21-22
Apolipoprotein A-I	6.8726	10.0452	6.4678	7.1802	-0.2724	A5-6
Cripto-1	6.2497	7.3180	5.3543	5.4354	-0.3224	B11-12

Adiponectin	7.2714	6.9070	4.6779	6.3473	-0.3563	A3-4
Complement Factor D	9.0969	9.9209	7.0896	7.3093	-0.3996	B7-8
Leptin	5.3909	8.5243	4.4610	5.8664	-0.4000	G1-2
MIP-3β	4.6019	6.5168	4.7185	2.8993	-0.4440	G21-22
CD14	13.8893	16.5246	9.0050	10.9085	-0.6121	A19-20
Cystatin C	37.4877	44.1524	26.1073	24.3430	-0.6807	B13-14
BDNF	60.0526	58.8622	34.2836	33.4567	-0.8119	A15-16
Myeloperoxidase	10.7519	6.9595	5.3059	4.4327	-0.8231	H1-2
BAFF	24.4597	27.4547	13.3103	14.0344	-0.9223	A13-14
Complement Component C5/C5a	12.4153	14.2965	5.3047	6.4296	-1.1893	A17-18
